# Diagnostic reference level curves for paediatric fluoroscopic imaging in the Netherlands

**DOI:** 10.1007/s00330-025-11643-9

**Published:** 2025-09-06

**Authors:** Goswin O. Croes, Ingrid M. Nijholt, Martijn F. Boomsma, Gitta Bleeker, Marcel J. W. Greuter, Cécile R. L. P. N. Jeukens, Carola van Pul, Jenny Siegersma, Geert J. Streekstra, Alie Vegter, Alida J. Dam-Vervloet

**Affiliations:** 1 Department of Medical Physics, Isala Hospital, Zwolle, The Netherlands; 2 Department of Medical Imaging, Radboud University Medical Center, Nijmegen, The Netherlands; 3 Department of Radiology, Isala Hospital, Zwolle, The Netherlands; 4 Department of Radiology, OLVG, Amsterdam, The Netherlands; 5 Department of Radiology, University Medical Center Groningen, University of Groningen, Groningen, The Netherlands; 6 Department of Radiology and Nuclear Medicine, Maastricht University Medical Center, Maastricht, The Netherlands; 7 Department of Medical Physics, Maxima Medical Center, Veldhoven, The Netherlands; 8 Department of Medical Physics, Martini Hospital, Groningen, The Netherlands; 9 Department of Biomedical Engineering & Physics, Amsterdam UMC, Amsterdam, The Netherlands; 10 Department of Radiology, Treant Zorggroep, Emmen, The Netherlands

**Keywords:** Paediatrics, Fluoroscopy, Diagnostic reference levels

## Abstract

**Objectives:**

Establishing paediatric DRLs is challenging due to sparse data availability. The objective was to assess paediatric fluoroscopic dose levels in Dutch clinical practice, as current diagnostic reference levels (DRLs) need updating following the European Guidelines on DRLs for Paediatric Imaging (PiDRL).

**Material and methods:**

Air Kerma-area Product (KAP) values were retrospectively collected from paediatric patients (0–18 years) who underwent fluoroscopic procedures in nine Dutch hospitals between 01-01-2017 and 01-06-2021. The protocols included were: micturating cystourethrography (MCU), upper gastrointestinal (Upper GI) and lower gastrointestinal with contrast enema (Lower GI). In accordance with the PiDRL recommendations for sparse data, the 75th percentiles of the median KAP values per age group from each hospital were fitted using an exponential dose-age curve, resulting in an age-dependent DRL curve. DRL values were compared to Dutch, other European national and European DRLs.

**Results:**

A total of 971 examinations were included. For MCU, the proposed DRL curve was lower than the Dutch DRLs. The proposed DRL curve for Upper GI was also lower than the UK DRLs. No DRL curve could be established for Lower GI due to limited data.

**Conclusions:**

Paediatric fluoroscopic dose levels in this study are substantially lower than the current Dutch DRLs, indicating the need for new national Dutch DRLs for MCU. This study proposes using a DRL dose-age curve to update Dutch paediatric DRLs. Using the proposed curve method, more DRLs could be established than with the conventional method. The proposed DRL curves might serve as input for updating Dutch paediatric DRLs.

**Key Points:**

***Question*** *Current Dutch diagnostic reference levels for paediatric fluoroscopy need updating, which is challenging due to sparse data availability.*

***Findings*** *Observed dose levels in this retrospective study indicate a potential decrease in Dutch diagnostic reference levels for paediatric fluoroscopy, using a dose-age curve method.*

***Clinical relevance*** *Updating Dutch paediatric fluoroscopic DRLs using a dose-age curve method allows for the establishment of DRLs in case of sparse data availability. This allows for further optimisation of radiation doses in the paediatric population.*

**Graphical Abstract:**

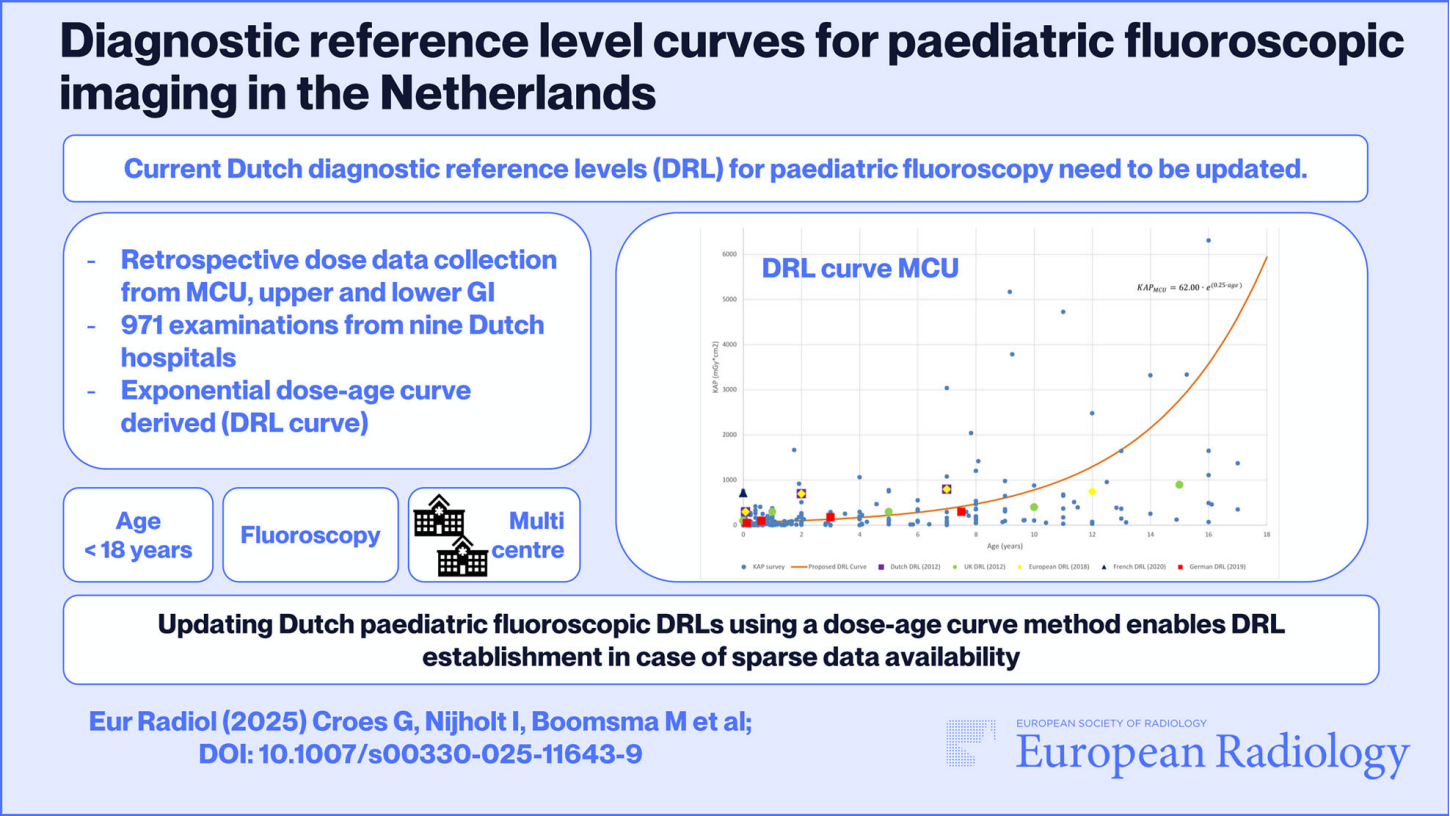

## Introduction

As the paediatric population is known to be considerably more sensitive to the carcinogenic effects of ionising radiation than the adult population [1, 2], it is crucial to optimise radiation doses for medical imaging in the paediatric population. Fluoroscopy is an important source of ionising radiation exposure in the paediatric population, but remains a valuable paediatric imaging tool due to its widespread availability in hospitals.

A well-established method to optimise radiation doses in medical imaging is diagnostic reference levels (DRLs), as introduced by the International Commission on Radiological Protection in 1996 [3–6]. DRLs are indicative radiation doses for standard diagnostic medical imaging procedures and serve as a framework to identify outlier doses and trigger a local review if they are consistently exceeded [7]. A recent commentary advocates for the use of DRLs as a dynamic optimisation tool that follows technological and clinical advances [8]. Since the European Guidelines on DRL for Paediatric Imaging (PiDRL) [9] were published in 2018, national paediatric DRLs for fluoroscopy procedures have been published for the UK, France and Germany [10–12].

In 2013, DRLs were established for the Netherlands [13] as required by European and Dutch regulations [14, 15]. Due to a lack of paediatric data for fluoroscopy, only Dutch paediatric DRLs for micturating cystourethrography (MCU) were established for age groups < 5 years (KAP values: 300 mGy·cm^2^ (0 years), 700 mGy·cm^2^ (1 year) and 800 mGy·cm^2^ (5 years)), based on UK DRLs from a review conducted in 2000 [13, 16]. However, the PiDRL guidelines [9] recommend the development of DRLs for three different paediatric fluoroscopic protocols: micturating cystourethrography (MCU), upper gastrointestinal (Upper GI) and lower gastrointestinal with contrast enema (Lower GI) and five different age groups (0 < 1 month; 1 month < 4 years; 4 < 10 years; 10 < 14 years; 14 < 18 years). Furthermore, the PiDRL guidelines recommend updating national DRLs every 5 years [9]. Therefore, there is an urgency to establish updated Dutch DRLs for fluoroscopic procedures in children.

One of the main challenges in establishing paediatric DRLs is the large variation in length and weight between different age groups, which leads to large variations in dose levels. Another challenge is the sparse availability of paediatric dose data. Establishing DRL dose-weight or dose-age curves [17–19] as opposed to the conventional method where a DRL per age group is established [9], overcomes these challenges and allows paediatric fluoroscopy procedures to be compared to the DRL at any age.

Our aim was to assess paediatric fluoroscopic dose levels based on exposures in Dutch clinical practice to update and extend the Dutch paediatric fluoroscopic DRLs (MCU, Upper GI, Lower GI), based on PiDRL recommendations and the use of a DRL curve.

## Material and methods

### Data collection

Between April and June 2021, a national survey was conducted by the Dutch PiDRL working group with the support of the Dutch Radiological Society (NVvR), Dutch Society of Medical Imaging and Radiotherapy (NVMBR) and the Dutch Medical Physics Society (NVKF). The PiDRL working group invited all 65 Dutch radiology departments performing both adult and paediatric procedures from academic and general hospitals to participate in the survey. The survey consisted of three parts: plain radiography, fluoroscopy, and CT. In this study, we will focus on the fluoroscopy data. Dutch radiology departments were asked to send retrospective data on paediatric (0–18 years) fluoroscopic procedures as recommended by the PiDRL guidelines [9]: micturating cystourethrography (MCU), upper gastrointestinal (Upper GI) and lower gastrointestinal with contrast enema (Lower GI). The MCU procedures include protocols such as bladder, micturating cystography, micturating cystogram and urethrogram. The Upper GI includes protocols such as stomach, oesophagus, swallow and stomach/duodenum. For an overview of the equipment, see Table 1.

**Table 1:** Equipment overview

Hospital	Hospital type	Vendor	Type
A	General	Philips Medical Systems	MultiDiagnost Eleva
B	General	Philips Medical Systems	MultiDiagnost Eleva
C	Academic	Siemens	AXIOM-Artis
D	Academic	Philips Medical Systems	MultiDiagnost Eleva
E	Academic	Siemens	AXIOM-Artis
F	Academic	Siemens	Artis Zee
G	General	Philips Medical Systems	CombiDiagnost (R90)
H	General	Philips Medical Systems	MultiDiagnost Eleva
I	General	Philips Medical Systems	MultiDiagnost Eleva

Hospital (anonymised), Hospital type (general or academic), Vendor and Type (equipment)

Air kerma-area product (KAP) values were retrospectively collected from paediatric patients who underwent a fluoroscopic examination between 1-1-2017 and 1-6-2021.

Inclusion criteria were:

- Patients aged less than 18 years
- Examinations with a KAP value greater than 0 and with KAP information available
- Hospitals with more than 10 KAP values for each examination type
- Examination protocols: MCU, Upper GI and Lower GI

### Data analysis

KAP values of all image acquisitions per examination were summed for each patient.

We calculated paediatric DRLs using two methods as recommended by the PiDRL guidelines:

1. The conventional method is based on the KAP values per discrete weight/age group as defined in the PiDRL guidelines
2. A curve method in the case of sparse data based on local DRL curves of each individual hospital

All analyses were conducted in R (R Core Team 2022) [20] and in Microsoft Excel (version 16).

Method 1: This is the method recommended by the European PiDRL guidelines [9]. According to these guidelines, only data from hospitals that provided data of more than 20 examinations for a given fluoroscopy protocol in each age group (0 < 1 month; 1 month < 4 years; 4 < 10 years; 10 < 14 years; 14 < 18 years) were used. The median (Q2) of the KAP was determined for each fluoroscopy protocol per age group per hospital. The DRL for dose was then calculated as the 75th percentile (Q3) of the medians of all hospitals per age group.

Method 2: This method consists of three steps:

- A quantile regression [21] based on an exponential trend line including intercept was applied to the dose-age data of each hospital separately to estimate its 50th percentile dose curve. For this method the restriction in method 1 to exclude data based on less than 20 patients was not applied.
- Transformation of a continuous curve into discrete data points with a sampling rate of 0.01 years along the *x*-axis (representing age). These curve-derived values were obtained from the separate 50th percentile dose curves per hospital.
- Quantile regression [21] was applied to the newly constructed dataset using the discrete data points of all hospitals to generate a 75th percentile curve representing the DRL curve. In generating the regression curve, data points were weighted with a weighting factor (*Wf_L*) to account for differences in data contributions across hospitals. The weighting factor was determined for each hospital and fluoroscopy protocol based on the number of procedures (*Np*) performed and the data collection period (*tf*), defined as the duration over which the procedures were performed. The weighting factor was determined according to the following equation:

1 $${{Wf}\_L}_{{hospita}{l}_{A}}={{Np}}_{{hospita}{l}_{A}}/{{tf}}_{{hospita}{l}_{A}}$$

The results of method 2 were compared to existing Dutch and available national DRLs of other European countries. The DRL of the MCU was compared to the Dutch DRL [9, 13]. For all protocols, a comparison was made to available European, UK, French and German DRL [6, 9, 11, 12]. Furthermore, a comparison was made between the two methods, 1 and 2. To this extent, discrete DRL values were determined by using the dose-age curve established by method 2 to calculate the median DRL value per age group.

## Results

Dose data were collected from seven Dutch hospitals in response to the survey (four academic and three general), with a total of 971 examinations (Table 2). The DRL for MCU could not be calculated for age groups ≥ 10 years for method 1, as the available data was less than 20 examinations per hospital (see Table 2). However, method 2 could be applied to all age groups for MCU. For Upper GI, the DRL was calculated according to both methods. For the Lower GI protocol, no DRL for methods 1 and 2 was calculated because the data included only two hospitals.

**Table 2:** Data survey

Protocol	Age group	NH	NE	KAP (mGy•cm^2^)						
				DRL method 1	DRL method 2	Dutch DRL (2012)	European DRL (2018)	UK DRL (2012)	French DRL (2020)	German DRL (2019)
MCU	0–< 1 month	6	80	30	62	300 (0 years)	300 ( < 5 kg)	100 (0 years)	720	50 (0–< 3 m)
	1 month–< 4 years	6	163	87	104	700 (1 year)	700 (5–< 15 kg)	300 (1 year)	n.a.	100 (3–< 12 m)
	4–< 10 years	6	64	309	366	800 (5 years)	800 (15–< 30 kg)	300 (5 years)	n.a.	180 (1–< 5 years)
	10–< 14 years	5	20	n.a.	1298	n.a.	750 (30–< 50 kg)	400 (10 years)	n.a.	300 (5–< 10 years)
	14–< 18 years	4	12	n.a.	3578	n.a.	n.a.	900 (15 years)	n.a.	n.a.
*Total*			*339*							
Upper GI	0–< 1 month	6	36	89	101	n.a.	n.a.	200 (0 years)	n.a.	n.a.
	1 month–< 4 years	7	228	102	137	n.a.	n.a.	400 (1 years)	160	n.a.
	4–< 10 years	7	120	278	195	n.a.	n.a.	500 (5 years)	n.a.	n.a.
	10–< 14 years	7	57	1,587	637	n.a.	n.a.	1800 (10 years)	n.a.	n.a.
	14–< 18 years	7	65	1,587	1,178	n.a.	n.a.	3000 (15 years)	n.a.	n.a.
*Total*			*506*							
Lower GI	0–< 1 month	2	16	n.a.	n.a.	n.a.	n.a.	n.a.	290	n.a.
	1 month–< 4 years	2	69	n.a.	n.a.	n.a.	n.a.	n.a.	360	n.a.
	4–< 10 years	2	23	n.a.	n.a.	n.a.	n.a.	n.a.	n.a.	n.a.
	10–< 14 years	1	6	n.a.	n.a.	n.a.	n.a.	n.a.	n.a.	n.a.
	14–< 18 years	2	12	n.a.	n.a.	n.a.	n.a.	n.a.	n.a.	n.a.
*Total*			*126*							
Total all protocols			971							

KAP values: DRLs according to method 1 and method 2 for the median value per age group. Dutch DRL (2012), European DRL (2018), UK DRL (2012), French DRL (2020) and German DRL (2019) are shown with their respective age values or weights in brackets. For the Lower GI protocol, no DRL could be calculated due to insufficient data

*MCU* Micturating cystourethrography, *GI* Gastrointestinal (tract), *KAP* air kerma-area product, *DRL* diagnostic reference level, *n.a.* not available

Number of hospitals (NH), number of examinations in the study (NE)

**Fig. 1:**
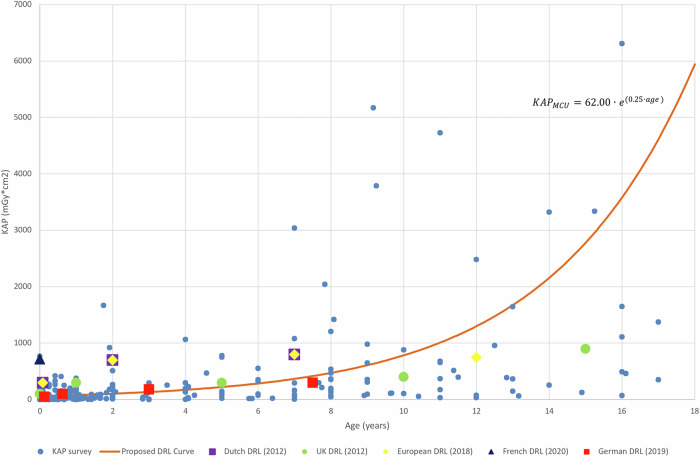
Diagnostic reference level (DRL) curve method 2 for micturating cystourethrography (MCU), showing air kerma-area product (KAP) values from surveyed hospitals and the proposed DRL curve (including equation) alongside Dutch, UK, European, French and German DRLs

**Fig. 2:**
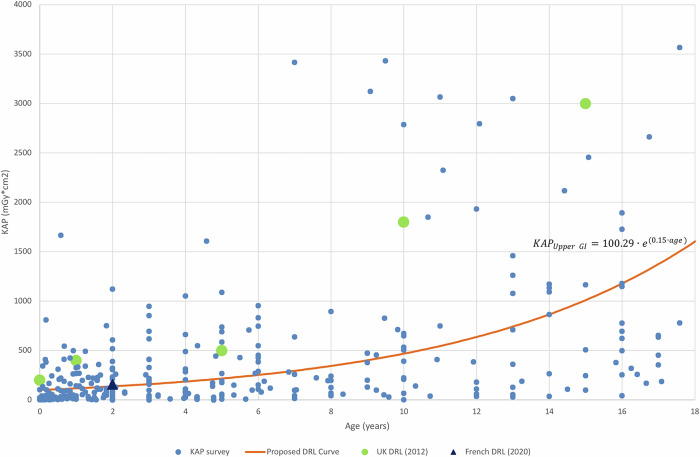
Diagnostic reference level (DRL) curve method 2 for upper gastrointestinal tract (Upper GI), showing air kerma-area product (KAP) values from surveyed hospitals and the proposed DRL curve (including equation) alongside UK, and French DRLs

As the paediatric patient gets older, the median KAP values and DRL values for all fluoroscopic examinations showed increasing dose levels (Table 2; Figs. 1 and 2).

48% of the data was provided in the age group 1 month < 4 years for MCU and 45% of the data was provided in the age group 1 month < 4 years for Upper GI.

For the MCU, the discrete DRL curve values were lower than the current available Dutch DRLs. In comparison with other (national) DRLs, the discrete DRL curve values were lower than European DRLs except for one age group (10–< 14 years); lower for the age groups of < 4 years compared to national UK DRLs and higher for the age groups of ≥ 4 years; lower compared to the national French DRL for the available age group; comparable with the age groups of < 4 years and higher than the other age groups of ≥ 4 years for the national German DRLs [6, 9, 11, 12].

For Upper GI, the discrete DRL curve values were lower than the current UK DRLs for all age groups and comparable with the French DRL for age group 1 month–< 4 years [6, 11].

## Discussion

The main purpose of this study was to assess Dutch paediatric fluoroscopic dose levels in clinical practice in order to update and extend Dutch national paediatric fluoroscopic dose levels for MCU, Upper GI and Lower GI, as recommended by the PiDRL guidelines and based on a curve method. Two methods, a conventional (method 1 in our study) and curve method (method 2 in our study), were applied for calculating the DRLs for MCU, Upper GI and lower GI. This study has shown that, according to the conventional method, DRLs could not be established for all age groups and protocols. Using the curve method, a DRL curve can be determined for MCU and Upper GI for all age groups. The PiDRL task force has recommended using the curve method. For the lower GI, neither method could be used due to insufficient data (only two hospitals).

Because children differ in terms of height and weight, a division into categories is necessary for the conventional method. The method for determining national paediatric DRLs is not very uniform in terms of the classification of these categories [6, 11, 12]. The PiDRL guidelines recommend weight categories due to better correlation with dose values. But the PiDRL guidelines also indicate that an age-based category classification can be used temporarily until weight-based dose collection is possible. Although weight is the recommended patient characteristic to classify body examinations, only two publications report DRLs in weight classes [11, 12]. Weight categories are determined based on age. The PiDRL guidelines provide a relationship between weight categories and age, based on a correlation between average weight per age. Because patient weight is often unavailable, this study proposes the use of age to categorise, which can be easily found in the electronic medical record and dose management system. By choosing an age-based curve method, the assessment becomes relatively easy to implement for hospitals.

A strength of the study is the determination of DRLs for the upper GI protocol based on recent Dutch data, which were previously unavailable. Additionally, the DRLs for the MCU protocol were updated using recent data, replacing outdated information. For the lower GI protocol, however, these procedures are not often performed; as such, the data provided was insufficient to calculate a dose-age curve. The observed dose levels were lower than the national DRL for MCU. Compared to the European DRLs and other national DRLs, this study shows a mixed pattern: some DRL values were lower, some were comparable, and others were higher across different age groups for both MCU and Upper GI. Lower dose levels can be attributed to dose optimisation and mandatory DRL testing [15] and to technological advances and protocol optimisation such as the use of pulsed fluoroscopy, limited use of magnification, proper dose selection, appropriate grid use, proper filtration, and the last image hold feature [22, 23]. The variation in DRLs across European countries can be attributed to several factors such as differences in clinical practice, patient population characteristics and outdated equipment.

The limitations of our study are mainly related to the small number of hospitals that responded to the survey. Furthermore, a small number of KAP survey data was provided (e.g., the data of the MCU procedure for the age > 14–18 years is only 12 exams). Most of the data was provided in the age group 1 month < 4 years. Different imaging protocols are tailored not only to specific anatomical regions but also to age-related pathologies and the diagnostic needs of each clinical indication, e.g., paediatric imaging for conditions like vesicoureteral reflux (VUR) are more common in younger patients up to two years, whereas older children are often imaged in a trauma setting. The curve can also be improved by increasing the sample size. As the sample included four academic hospitals and three general hospitals, this study considered the hospitals included in the survey to be representative of all Dutch hospitals. A limitation of the curve method is that it is derived from an exponential function based on pooled data, where relatively greater weight is assigned to the DAP survey values for ages up to 10 years. For ages above 10 years, the smaller sample size results in less accurate estimations of the input curves of the individual hospitals (which contribute as input to the pooled data). The exponential function was chosen for its simplicity and ability to capture dose-age trends. However, in fluoroscopic procedures, where KAP is less strongly linked to patient size compared to modalities like plain X-rays, alternative models might provide a better fit. Future studies should explore these options to improve the accuracy of age-dependent DRL curves for paediatric fluoroscopy. Another potential limitation of this study is the reliance on KAP values from participating hospitals without explicitly verifying the calibration accuracy of their fluoroscopy equipment. While routine calibration is standard in the Netherlands [24], variations in calibration status could affect the accuracy of the derived DRL curves [25]. Future studies should consider verifying equipment calibration, though this may be challenging due to time constraints.

An observation is the variation in dose levels among different age groups. Sources of variation are related to different factors such as patient size, clinical indication, anatomic region and imaging protocols. Another observation is the large variation in dose levels for the same fluoroscopy protocol between hospitals. A recent study reported that the distribution of dose levels observed in fluoroscopy-guided interventional procedures of the same type can be very large due to technique variations, operators’ experience and, most importantly, differences in the intrinsic complexity of the procedure [8]. The large variation also emphasises the need for operators to be aware of ongoing dose optimisation and timely replacement of outdated equipment.

Periodic updating and extension (e.g., lower GI) of the DRL for the paediatric population remains important and should be performed every 5 years [9]. Currently, the data in this study was collected both manually and automatically with a dose monitoring system. As we move further towards periodic updating, automatic data collection for this task seems mandatory, as data is readily available. This can be achieved by installing local dose monitoring systems, which enable automatic collection of dose level data and automatic comparison with national DRLs.

## Conclusion

Our study showed that the current dose levels for paediatric fluoroscopic imaging are substantially lower than the current Dutch DRLs for MCU. For the Upper GI protocol, a Dutch DRL is not currently available. The proposed curve method allows for the establishment of more DRLs compared to the conventional PiDRL method. Therefore, our data support the validity of the curve method, which implies that new Dutch national DRLs for MCU and Upper GI should be established on the curve method. The proposed DRL curves might serve as input for the establishment of updated Dutch paediatric DRLs.

## Abbreviations

DRL: Diagnostic reference level
GI: Gastrointestinal (tract)
ICRP: International Commission on Radiological Protection
KAP: Air Kerma-area Product
MCU: Micturating cystourethrography
PiDRL: European Guidelines on DRLs for Paediatric Imaging
