# Correction: Diagnostic reference level curves for paediatric fluoroscopic imaging in the Netherlands

**DOI:** 10.1007/s00330-025-12216-6

**Published:** 2026-02-04

**Authors:** Goswin O. Croes, Ingrid M. Nijholt, Martijn F. Boomsma, Gitta Bleeker, Marcel J. W. Greuter, Cécile R. L. P. N. Jeukens, Carola van Pul, Jenny E. Siegersma, Geert J. Streekstra, Alie Vegter, Alida J. Dam-Vervloet

**Affiliations:** 1 Department of Medical Physics, Isala Hospital, Zwolle, The Netherlands; 2 Department of Medical Imaging, Radboud University Medical Center, Nijmegen, The Netherlands; 3 Department of Radiology, Isala Hospital, Zwolle, The Netherlands; 4 Department of Radiology, OLVG, Amsterdam, The Netherlands; 5 Department of Radiology, University Medical Center Groningen, University of Groningen, Groningen, The Netherlands; 6 Department of Radiology and Nuclear Medicine, Maastricht University Medical Center, Maastricht, The Netherlands; 7 Department of Medical Physics, Maxima Medical Center, Veldhoven, The Netherlands; 8 Department of Medical Physics, Martini Hospital, Groningen, The Netherlands; 9 Department of Biomedical Engineering & Physics, Amsterdam UMC, Amsterdam, The Netherlands; 10 Department of Radiology, Treant Zorggroep, Emmen, The Netherlands

**Correction to: European Radiology**

10.1007/s00330-025-11643-9

Authors’ second initials have been added back for indexing reasons.

The original article has been corrected.

